# Re-visiting systematic observation: A pedagogical tool to support coach learning and development

**DOI:** 10.3389/fspor.2022.962690

**Published:** 2022-08-23

**Authors:** Ed Cope, Christopher J. Cushion, Stephen Harvey, Mark Partington

**Affiliations:** ^1^School of Sport, Exercise and Health Sciences, Loughborough University, Loughborough, United Kingdom; ^2^The Gladys W. and David H. Patton College of Education, Ohio University, Athens, OH, United States; ^3^Department of Sport and Physical Activity, Edge Hill University, Ormskirk, United Kingdom

**Keywords:** systematic observation, coach development, pedagogy, coaching, learning

## Abstract

Systematic observation has been one of the most employed data collection methods in sport coaching literature. Initial work, originally undertaken in the 1970's, and gaining traction in the 80's and 90's looked to predominately offer descriptions of coaches' behavior. While this research continues to offer a significant contribution to the fields understanding of what coaches do during practice, systematic observation used only in this way has unfulfilled potential. The premise of this paper is to consider systematic observation as a coach development tool—a precedent which has been set in the literature. The arguments made are based on an alternative way of thinking about systematic observation, as a pedagogical tool that supports coaches in better understanding themselves and their pedagogical practice. Principles of dialogic pedagogy are used as the basis of our argument whereby “researchers” and “coaches” work collaboratively to co-construct knowledge and support coach reflection, and ultimately develop coaches' practice.

## Introduction

A desire by early coaching scholars for some dispassionate base-line data to find out what (good) coaches do (Cushion, 2010), has grown to become a significant focus within sport coaching research over the last 40 years (Gilbert and Trudel, 2004; Cushion et al., 2012a; Cope et al., 2017). Since the pioneering work of Tharp and Gallimore with legendary coach John Wooden (Tharp and Gallimore, 1976) to systematically identify coach behavior using a descriptive-analytical system, the systematic observation literature has evolved identifying coaches' behavior in training (and less so competition) across a diverse range of sports and sporting contexts (e.g., Lacy and Goldston, 1990; De Marco et al., 1996; Cushion and Jones, 2001; Potrac et al., 2007; Ford et al., 2010). The initial rationale behind this work remains as valid today as it did in the 1970s, in that coaches are central to the coaching process and what they say and do continues to impact performers' achievement and wellbeing. Therefore, understanding which behaviors translate into positive experiences and functioning on the part of athletes is critical for researchers and practitioners alike (Cushion, 2010). This potential for, and development of, systematic observation in coaching was greeted with considerable enthusiasm with many authors stressing its importance in establishing an empirical base for a “science” of coaching particularly related to coach behavior (e.g., Lacy and Darst, 1985; Lacy and Goldston, 1990; Seagrave and Ciancio, 1990). Indeed, this research was perceived as ushering sport pedagogy into “an era of legitimacy, innovation, and unparalleled activity” (Kahan, 1999, p. 18).

Since its inception, systematic observation research has provided coaching with a rich and promising literature, as well as a sound basis for studying coach behaviors, practice, and their relationship (Ford et al., 2010). Useful descriptions of coach behavior do exist, and what this body of work has done is to identify (within constraints) “tried and tested coaching behaviors” (Douge and Hastie, 1993, p. 54). More recently, systematic observation has been identified as an important and necessary part of understanding how coaches support their athlete's learning and development (Cushion et al., 2012b; Cope et al., 2017). For example, the use of systematic observation to identify coaches' behavior has enabled researchers to make links between what coaches do and the impact on athlete outcomes, including their motivation to participate (Lefebvre et al., 2021) and skill development (Smith and Cushion, 2006). In turn, recommendations regarding the potential benefits and drawbacks of using certain coaching behaviors over others in the pursuit of identifying positive athlete outcomes have ensued. For example, the potential of coach questioning in developing players decision-making and thinking skills has been highlighted (O'Connor et al., 2022), while Mason et al. (2020) studied coaches' use of feedback and identified this behavior as crucial to supporting athlete learning. However, in both cases, the employment of these behaviors does not automatically lead to such positive outcomes being realized. This is highlighted in Hattie and Timperley's (2007) review of feedback, where they identified certain forms of feedback (i.e., praise) having detrimental effects on learning. Equally, Cope et al. (2016) critically evaluated academy football coaches' questioning strategies and found that while coaches asked a relatively high number of questions, most of these did not require players to think critically about their performance. In all cases, systematic observation data has been a starting point to explore these and other individual behaviors in greater detail.

Perhaps most importantly, scholars identified the potential of systematic observation as a tool to support coach development. For example, work by Krane et al. (1991), De Marco et al. (1996), and Kidman (1997) all argued that descriptive-analytical systems provide data that can be used to increase coach awareness and change coach behaviors through coach self-assessment. That is, the ability to objectively evaluate and change one's own coaching performance. These authors argued that systematic observation as part of collaborative action research (CAR) can support coaches' self-directed training that combine video, analysis of coach behaviors, systematic observation data, feedback, and reflective questions—an approach adopted in more recent research (e.g., Gallo and De Marco, 2008; Partington et al., 2015; Cope et al., 2021). This involves using systematic observation to provide an initial analysis of what coaches are doing, and how this is aligned with coaches' practice intentions. Then, what follows is continued observation and reflective conversations with coaches, to support understanding and developments with practice. An iterative process is pursued exploring the relationship between coaches' behaviour, their development needs and their coaching practice. The evidence from this work has shown that this process using systematic observation data at its center alongside other pedagogical tools can elicit significant behavioral change (e.g., Partington et al., 2015; Cope et al., 2021).

Despite longstanding and clear guidance, and evidence of success, application of systematic observation as a tool to support coach development has remained limited. Indeed, systematic observation has a significant but unfulfilled potential in supporting coach education and development. Moreover, as our understanding of coaching has become more sophisticated, and research priorities have changed, despite its original popularity, systematic observation as a tool has fallen into disrepute and largely been dismissed. For reasons explored in this paper, coaching has disregarded existing accumulated knowledge rather than considered ways to integrate new approaches with what is already known (Cushion, 2010; Cushion et al., 2012a). Importantly, it is in the interests of coaching, and its development, that attempts are made to integrate existing work and ideas into a more sophisticated approach. To that end, there are opportunities to re-visit a systematic observation method and extend it beyond describing coaches' behavior. Re-visiting and using systematic observation can help formulate methods for practice and change and monitor pedagogical applications for coaches. This enables coaches to recognize patterns of behavior which would otherwise go unnoticed and give conscious attention to issues relating to pedagogical practice and practice design (cf. Kidman, 1997; Partington et al., 2015). To do this requires a repositioning of a systematic observation method and methodology away from collecting data *on* coaches toward using this method *alongside* coaches—where the coach becomes the researcher as well as the researched (Nind and Lewthwaite, 2018).

The purpose of this paper is to argue for revisiting systematic observation and how this method could be used as a pedagogical device to support coach development. The aim is to present the case for it as a pedagogical tool that “teaches” (Nind and Lewthwaite, 2018) coaches about themselves and their practice, and not just a means by which to identify and describe coaching behaviors. The significance of this work lies in providing a route to a method that supports coaches in thinking more reflexively about their practice, and in turn developing their pedagogical practice. This is important as it has been suggested reflexive thinking is critical to making “hard to reach” beliefs and assumptions more explicit and conscious, which in turn, is the starting point to enabling sustained changes to practice (Cunliffe, 2004; Cushion, 2013; Cope et al., 2017). This work carries additional significance in its consideration for how methods traditionally associated with research *on* coaches, can be used as methods *with* coaches to support their learning and development, while capturing the process.

## Systematic observation and sport coaching

Before discussing systematic observation as a device to support coach development, it is important to provide a brief account of how systematic observation is positioned and viewed in the existing literature. The premise of this discussion is to provide an account that addresses the criticism and reasons for systematic observation falling out of favor. Without this understanding and addressing the issues surrounding systematic observation, any attempt to promote the method will likely be unsuccessful.

Coaching research has traditionally been located within a dominant psychological discourse, which in turn, has its epistemological roots in the positivistic natural sciences (Ward and Barrett, 2002; Cushion, 2010) within a process-product paradigm. An assumption of which is that coaching behavior is unambiguous and there is a universal set of behaviors that coaches can transfer from one context to another and achieve the same results (Wragg, 1999). The paradigmatic roots of systematic observation have meant that its findings have tended to “reduce” and over-simplify the nature of coaching and homogenize coaching contexts (e.g., Cushion and Jones, 2001; Ford et al., 2010). Indeed, behavioral assessment has been criticized for being too simplistic, failing to embrace the complexity of the coach, both as a person and with how he or she engages with the wider pedagogical process. However, it has of course long been recognized that coaching behaviors *per se* do not stand alone as predictors of effective coaching (Douge and Hastie, 1993) nor do they “embrace the entirety of the coaching process” (Lyle, 1999, p. 14). While systematic observation of coaches has resulted in a wealth of information about coach behavior, observation instruments are limited in that they only measure direct styles of coaching, and used alone, stop short of accounting for context (Kahan, 1999; Cushion et al., 2012a).

To better understand the nature of the coaching process, coaching research has more recently focused on the social world of individual coaches, the interpretations of their experiences and the processes by which meanings and knowledge are used to guide actions (Potrac et al., 2002, 2007). This has resulted in mixed methodologies being employed (e.g., Potrac et al., 2002, 2007; Harvey et al., 2013), sometimes with systematic observation combined with interviewing revealing much about coaches' cognitive strategies and the context of behavior (Cushion et al., 2012a), assisting coaches in reflecting on their coaching (Partington et al., 2015), and providing opportunities to discuss coach prior learning, athlete characteristics and environmental conditions (Cushion et al., 2012b). Despite this, and its original popularity, in more recent times systematic observation methods and the resultant findings have fallen out of favor (Cope et al., 2017). Many authors have tended to highlight their weakness (e.g., Abraham and Collins, 1998) as well as favoring other usually more qualitative methods, namely interviews with coaches (Potrac et al., 2007). Referring to earlier systematic observation work, Abraham and Collins (1998) rightly questioned the “absolute” interpretations made from data generated (i.e., using behavior X leads to outcome Y) rightly, coaches' behavior and practice cannot be reduced to such simplicity. These criticisms of positivist informed approaches, has set the scene to usher in and nurture alternative paradigms in the study of sport coaching (Lyle and Cushion, 2017). These are largely based on a shift to understand the interplay between coach, athlete, and environment (Cushion, 2010) alongside a move to a more interpretive and subjective view of the nature of coaching and coaching experience, resulting in coach behavior analysis being portrayed as “superficial”.

The examination of the pedagogical strategies used by coaching practitioners is certainly a feature of coaching which benefits from different modes of inquiry. Importantly though, advocates of such an approach (e.g., Potrac et al., 2002, 2007; Cushion, 2010) never suggested that systematic observation methods should be “dropped”. Rather, that the systematic observation of coaches should be built on, followed up, and supported with additional methods such as interviews and/or participant observation work. Methods in themselves are neither inherently good or bad but rather more or less appropriate based on the question being asked and what is being investigated (Morgan, 2007). So, systematic observation is not necessarily better or worse than any other method. However, if the aim is to identify what coaches do when coaching, providing relatively objective data that helps them understand and develop their coaching, systematic observation is an important “tool” in helping achieve this aim.

Combining systematic observation with other methods enables a deeper understanding of the multifaceted interactions involved in the dynamic coaching process, an awareness of the contexts in which coaches act, and the influence these contexts have upon their respective pedagogical strategies (Potrac et al., 2002, 2007; Cushion et al., 2012b). While coach behavior analysis may be portrayed as “superficial” in one sense, we still know relatively little about coaches' practice, or the impact of education and development on coaching practice (Lyle, 2002; Potrac et al., 2007; Cushion, 2010; Cushion et al., 2012a; Stodter and Cushion, 2017, 2019; Cope et al., 2021). Examining what coaches do and why they do it, still offers much in developing our understanding about coaching and informing coach development. Systematic observation data are crucial in the process of finding out what coaches do when they coach, because these data are not reliant on coach recall or perceptions in a way other methods (i.e., interviews, surveys) are. It is identifying objective coaches' behavioral profiles that provides the basis from which changes can be made (Cushion et al., 2012a).

Perhaps the most damning for systematic observation use is the lack of perceived “fit” with the current interpretive assumptions underpinning much coaching research. Unfortunately, systematic observation has fallen foul of the conflation of paradigm with method common in coaching research. More specifically, systematic observation's association with behaviorism and its tenets (Peel, 2005). However, these associations speak more to how the method is used and data reported rather than the method itself. That is, aggregation of coaches' behavioral data, inter-individual/group comparisons, and the ironing out of context. Indeed, while paradigm does not define method, the association of systematic observation with positivist assumptions along with a decline in the sport coaching literature focusing on coaches' practice has seen an attendant diminution in the use of systematic observation and a rise in retrospective analysis, typically using qualitative interpretive methods. However, systematic observation is no less “interpretative” if the data are used intra-individually, alongside consideration of context and not used to make “generalisable” cause and effect claims.

Taken together, it remains not only necessary to record the pedagogical behaviors of coaches, but to also reflect upon the appropriateness of such behaviors for developing desired outcomes. Particularly as it is well-demonstrated that some coaches have limited awareness of how they behave in various ways, with athletes' ratings correlating more strongly with observed behaviors than the coaches' own self-ratings (e.g., Harvey et al., 2013; Partington and Cushion, 2013; Raya-Castellano et al., 2021). Low self-awareness among coaches is well-evidenced, coaches are unaware of their moment-to-moment behavior (De Marco et al., 1996). Retrospective studies reporting coaches' perceptions of their behavior, typically found through survey-based measures, do nothing to address these well-known coach tendencies to low self-awareness and the limitations of often overly optimistic self-report. This issue is not unique to coaching but has been found in other educational domains (i.e., Hook and Rosenshine, 1979), as well as physical activity settings (i.e., McKenzie, 2016). Any advances in coach education and development would likely be fruitless if coaches lack basic levels of self-awareness, particularly in practice environments driven by a strong sub-culture (Cushion et al., 2012b; Partington et al., 2015; Raya-Castellano et al., 2021).

Despite its limitations, systematic observation provides relatively objective descriptive data of “actual” (demonstrated in context) coaching behavior. As such, it provides a tangible anchor “for what coaches do” and is a precursor, or gateway, for the use of accompanying other methodologies (cf. Brewer and Jones, 2002; Gilbert and Trudel, 2004; Potrac et al., 2007; Cushion et al., 2012a), such as interviews with athletes and coaches. Given this, systematic observation has an important role to play in developing representative evidence-based foundations for coach development. Therefore, revisiting the role of systematic observation in researching coaches and coaching as support for coach development seems an obvious step. In the section that follow, we discuss how this can be delivered and supported.

## Revisiting systematic observation: A tool to support coach learning and development

If the possibility for systematic observation as a coach development tool is to be realized and gains a foothold beyond the few studies cited (i.e., Gallo and De Marco, 2008; Partington et al., 2015; Cope et al., 2021), a shift in how this method is used and reported is required. Because behavioral profiles are the dominant output of systematic observation these have often been emphasized at the expense of reporting how data are fed back to and used by coaches to inform their learning and development. This lack of focus on detailing the feedback process with coaches, suggests it is not happening or that it is not considered worthy of reporting. This could suggest coaches are not seen as a necessary or important part of this process and are positioned on the periphery of the research. It is important to remember that coaching practice is not a construct that is in some way subordinate to the needs of empirical work (Cushion, 2007, 2022). The relationship between research and practice, and researchers and coaches, needs to be further developed and understood. Indeed, coaching practice informed by research is worthy of further discussion.

There has been a concerted effort to better engage coaches in the research process through projects using forms of CAR (e.g., Curzon-Hobson et al., 2003; Evans and Light, 2007; Clements and Morgan, 2015; Chapron and Morgan, 2020; Cope et al., 2021). This research highlighted that engaging with coaches led to them developing their reflective capabilities, feeling an increased sense of motivation and autonomy, developing their coaching knowledge, and changing aspects of their coaching practice. While these findings are promising, there is a need to focus on the mechanisms (i.e., the methods) that bring about changes to any aspect of coaches and their practice. In other words, how can methods be thought about as a coach development device, rather than only as something that tells us about an element of coaching? More specifically, a dialogue needs striking with coaches that demonstrates an engagement and collaboration with their coaching practice (Cushion, 2010, 2022). It is here that we consider systematic observation has a significant role to play.

In the context of teacher education, Nind and Lewthwaite (2018) have called for a re-positioning of research methods using the notion “methods that teach”, to change the dynamic between researcher and participant, and the role the “method” is there to fulfill. Other writing in a teacher education context has warned against using research methods as ends in themselves but seeing the wider potential these hold in being a catalyst for teachers' learning and development (O'Leary and Price, 2016). This seems particularly relevant for observational methods, such as systematic observation, which can reveal aspects of a coach's practice that would otherwise remain unknown. Put another way, the behaviors coaches' employ are difficult to know without direct observation of their practice. Systematic observation is a method that captures these behaviors, but as importantly, these data can (and have) act as a stimulus for conversation (Cope et al., 2021). For example, these conversations are instigated by providing coaches' access to their behavioral profile linked to video footage. Coaches are then invited to discuss their practice, with the researcher/coach developer serving to support these discussions through supportive, reflective questioning. Indeed, it is the framing of these questions as much as their contents, which is important. A starting question might be “could you talk me through your interpretations of the data”? before moving onto more focused questioning, such as “there seems an emphasis on providing lots of general feedback, could you explain your thinking here”?

To genuinely re-position the role of methods requires a fundamental shift in the role of the researcher (Nind and Lewthwaite, 2018). Carroll (2009) uses the term “alongsider” to describe the process of researchers working with and alongside practitioners, thus placing a shared lens on each other's practice. In this way, the researcher and the participant would not exist in the traditional sense, but instead as both co-researchers and co-participants who work dialectically. Simply stated, the “researcher” supports the “participant” in their learning, but equally the “participant” helps the “researcher” better understand the effectiveness of their support (Cunliffe, 2004). This means knowledge is shared and developed collaboratively over an extended period (Nind and Lewthwaite, 2018), and through collaboration, these coaching conversations, become learning tools (Readman and Rowe, 2016). In practice, this might result in the researcher being invited by the coach to offer their interpretations of coach's behavior or discussing with coaches the relationship between the broader researcher evidence and the coaches' behavioral profile. The benefit of researcher and coach working dialectically are wide ranging and include supporting what is noticed (Amador et al., 2016), and developing a deeper sense of reflection (Stodter et al., 2021). However, as the CAR literature in coaching and other action research in related pedagogical contexts (i.e., Casey, 2012, 2013) has reported, researchers working in the ways described here are far from straightforward. As Casey et al. (2018) write, the challenge for researchers when supporting practitioners (i.e., a coach) is in how they move from an outsider of a practitioners' practice toward someone who is immersed within their practice—the insider-outsider dilemma is a considerable one to overcome.

Writing in a broader educational context, O'Leary (2020) suggests a problem with observation of teachers practice lies in its association with an assessment or judgement of teachers' practices. Viewing observation in this way has been suggested to create a “performative culture” and would potentially undermine any attempts to work collaboratively. Ball (2003, p. 216) defines performativity as “a technology, a culture and a mode of regulation that employs judgements, comparisons and displays as means of control, attrition and change”. Systematic observation research has sought to identify the behaviors of “expert” coaches (often defined by win-loss record), as a means of suggesting what effective coaching looks like (cf. Douge and Hastie, 1993). While certain coach behaviors and practice structures have been consistently identified in the literature, efforts to apply findings from an analysis of behavior or practice in prescriptive ways ignores the context under which those studies have been conducted (Douge and Hastie, 1993; Kahan, 1999). Positioning such findings as “effective coaching” has potentially led coaches to thinking there are universal “good” and “bad” coaching behaviors. So, some systematic observation studies have promoted certain behavioral profiles over others detached from context, setting the precedent of this method as performativity normalizing. Performativity has been promoted further through assessment processes as part of National Governing Body (NGB) qualifications. While distinct from systematic observation research, the pervasiveness of behavioral based competencies in coach certification (Cushion et al., 2021) remains, where coaches are required to meet prescriptive criteria to demonstrate competence often in contexts removed from those coached in (Chesterfield et al., 2010). In other words, coaches have had to coach in specific and formulaic ways to satisfy what was expected of them from an “assessor”.

Reverting to work of O'Leary (2020, p. 105), he argues that “the most important role observation has to play in education is not as a method of assessment but as a collaborative tool for exploring what, how and why we do what we do when teaching and learning”. Considering the application of systematic observation in coaching, this has only been partially achieved. This is not to say researchers have not engaged with coaches as part of the broader research process, but if it did happen the exclusion of any explanatory detail from methodology sections suggests something about how important this process was perceived. This point provides further support that the use of systematic observation has been a tool to collect data *on* coaches and not *with* them.

To help put the role of the coach at the center of their own learning, we consider the concept of dialogic pedagogy as useful in helping to think about the use of methods, such as systematic observation. Alexander (2008) identified four principles of dialogic pedagogy, which are (1) *collective*: teachers and students collaborate with each other to build knowledge and understanding, (2) *reciprocal*: teachers and students share responsibilities for the flow of discussion and consider alternative perspectives, (3) *supportive*: students voice their ideas freely within a constructive community and help each other to reach a common understanding, and (4) *cumulative*: teachers and students build on each other's ideas and chain them into a coherent line of inquiry. Furthermore, Mercer et al. (2010) characterize dialogic pedagogy as involving the “teacher” and “student” together in the generation and evaluation of ideas with a view of achieving explicit reasoning and the co-construction of knowledge.

Research over time has highlighted how coach education programmes based on principles like dialogic pedagogy, have resulted in positive coach outcomes, which include coaches experiencing a freedom to learn, feeling cared for, and developing more reflexive thinking (Krane et al., 1991; De Marco et al., 1996; Cope et al., 2021; Raya-Castellano et al., 2021). A reason for the reported positive coach outcomes were due to coaches feeling increased levels of agency in their own learning, something which has been reported in other educational contexts when a dialogic approach has been employed (Hennessy et al., 2017). In Cope et al.'s (2021) study, a systematic observation method was used to support coaches in identifying a coaching issue and to capture behavior change, and therefore offered some insight into its potential in supporting learning. In Raya-Castellano et al.'s (2021) work, systematic observation data served as a stimulus for engaging coaches in reflective practice, whereas in Krane et al.'s (1991) and De Marco et al.'s (1996) studies, systematic observation was used to support coaches in developing greater levels of self-awareness. In all these examples, using data grounded on coaches' behavior and practice was the starting point for dialogue. However, these studies, contrary to the position promoted in this paper have fallen short in detailing how systematic observation was used as a catalyst to promote dialogue between researcher and coach.

The process of starting a conversation is important, as according to Corlett (2012) once researcher and participant begin to interact and talk, the space for learning is created. Relatedly, Shulman (2000) discusses the idea of a scholarship of teaching, which is characterized by a presense of peer-review and critique, and an exchange of ideas between a professional community. Systematic observation has the potential as an iniating mechanism for a scholarship of coaching rather than to conclude a coach's behavior and practice. A critical factor in enabling a coach to talk is through supporting the “participant” to tell, and then re-tell experiences (Corlett, 2012). Data generated from systematic observation could play a critical role in this process because data are derived from coaches' practices, as opposed to coaches' perceived ideas about what they think happens when they coach. Research consistently demonstrates that showing and discussing with coaches' data pertaining to their behavior is a powerful learning experience, particularly when presented alongside video (Kidman, 1997; Partington et al., 2015; Raya-Castellano et al., 2021). A reason for this is that learners appear “struck” by what these combined data reveal, prompting critical reflexive thinking (Corlett, 2012; Partington et al., 2015; Hardman, 2020).

Some suggestions have been reported in the literature to help researchers think about systematic observation as part of a collaborative learning process with coaches, and thus support the reflexive thinking process. Twenty-five years ago, Kidman (1997) demonstrated that facilitated reflective processes allowed in-depth analysis of coaches' pedagogical skill and other dynamic behaviors specific to the coaching environment. While more recently, Raya-Castellano et al. (2021) showed that coach knowledge and awareness were developed mainly due to the clear opportunities for coaches to implement ideas and reflect on their delivery. The resulting reflection on behavioral data either reinforced coaches' delivery or enhanced their willingness to change. Either way, it seemed coaches having opportunities to practice in a supportive environment was a critical part of positioning systematic observation as a supportive, rather than performative process. In Stodter et al.'s (2021) study, reflective conversations were employed to support coaches in thinking about prevalent issues they wished to develop in their practice with the aim of this transferring into practice. Again, the starting point in this research was the coach and supporting them in reflecting on issues most pertinent to them. This is an alternative approach to coach development, which moves away from imposing ideas for the coach to work on related to their behavior and practice.

Learning takes time and sustained effort, and so for systematic observation to be positioned as a learning tool, the data generated must be presented and discussed between “researcher” and “coach” and/or coach and coach in an ongoing process. Doing so will support researchers and coach developers alike becoming immersed within the coaches' practice and developing a better sense of how they are attempting to coach. Partington et al.'s (2015) study is a good example of this, which showed behavioral change over a 3-year period with multiple touch points between the researcher and coaches as well as between coaches, with their behavioral data discussed in a number of different ways (i.e., one-to-one conversations, small group discussions). This study explains the need for trust and buy-in to be built with coaches before preceeding to support them in developing aspects of their behavior and practice. This is perhaps difficult to achieve given the limited number of observations and time observed in much of the systematic observation research (Cope et al., 2017).

A fundamental characteristic of dialogic practice is the agency of the “learner”. Positioning systematic observation as a learning tool through engaging coaches in dialogic practice is far more than presenting data multiple times and engaging in reflective conversations about these data. A critical part of systematic observation is validating that the behaviors and their definitions can be identified through observing coaches' practice (Brewer and Jones, 2002; Cushion et al., 2012a). To determine these, initial observation of practice takes place, and while coaches have been involved in the validation of these behaviors (Cushion et al., 2012a), often it is the researcher who selects what is observed and fed back. As part of a dialogic process, it would seem necessary for coaches to play a greater role in determining how the data generated from systematic observation is fed back to them. In turn, coaches should be better placed to understand the observation process and feel as though they can determine the direction of this ongoing process.

To work within the principles of dialogic pedagogy, coaches need to be involved in the entire design of the program of learning and in the ways in which methods such a systematic observation are used. If observational research is to serve as a means of affording “learners” the opportunities to inquire into their practice, then they must be involved in decisions regarding the observation template, what gets observed and how data are shared and fed back (O'Leary and Savage, 2020). This is not to say coaches' design the observation template *per se*, but rather the behavioral definitions are explained and feedback sought before observation commences. Coaches must also be supported in learning how data generated from systematic observation can be used to review and plan sessions both individually, but also as part of a wider coaching team. O'Leary and Savage (2020) found that when this happens, teachers focused on observational data to develop themselves and their practice, and not as a judgement of their practice. This in turn resulted in greater collaboration between the observer and observed as the power dynamic of the relationship had shifted.

## Moving forward with systematic observation

Systematic observation being used alongside qualitative methods has built on and extended the initial research undertaken, contributing to a greater holistic understanding of coaches' practice. Nonetheless, there is a need to revisit the use of systematic observation beyond its original conception. That is, as a method that identifies what coaches do as a basis for making claims about “effective” coaching, and later, as a method more concerned with supporting coaches' self-awareness and rationalization of practice. This means no longer confining systematic observation to research and researchers but connecting it with coaches to provide direct assistance in developing their coaching practice.

Systematic observation is one of coaching's most valuable tools and though not a panacea for all ills it has for several decades consistently proven its worth. It is a method worth revisiting and its use refined as a potential pedagogical tool that acts as a catalyst for coaches' learning and development. Used in this way, researchers take up the position as co-collaborator who work with coaches to support their learning akin to that of a coach developer, as opposed to a traditional relationship, which sees the researcher positioned as an impartial “other” who is collecting data *on* coaches. This is a fundamental shift in the role of the researcher and is not exclusive to the use of a systematic observation method. We encourage researchers to design studies using systematic observation in a way that thinks about its use as a coach development tool. However, we recognize that with this comes other requirements, such as a move beyond cross-sectional or “snap-shot” observations of practice over short time periods toward sustained use with coaches over longer periods (i.e., season and multiple season-long). By using the method in this way, the “researcher” and coach relationship can be built to enable an iterative process of observing and then reflective conversations to support a coach's development. However, as Stodter et al. (2021) point out, it requires a certain skill set to support yet understand when to challenge coaches' beliefs so that trust and respect is built with coaches. We accept this is not straightforward, and welcome research that explores coach developers' ability to connect with and support coach learning.

As part of revisiting the use of systematic observation, we also support coach developers and coaches themselves in playing a more active role in using this method as a pedagogical tool. We consider this important in helping turn coaches' attention toward their practice, and underpinning pedagogical knowledge and skills, as part of their planning and reflective processes. We accept that systematic observation instruments are not necessarily always user-friendly, and the time associated with analyzing behavior using the full versions of any systematic observation instruments is not always feasible or indeed necessary. Therefore, systematic observation instruments need refinement and calibration to make the methodology less restrictive and more appealing, and something coaches can engage with autonomously of “researcher” or “coach developer”. Indeed, both Cushion et al. (2012a) in coaching and Hennessy et al. (2016) in teacher professional development, make the case for not using the full version of a systematic observation instrument for professional development purposes, but it should be noted this is after a baseline behavioral profile has been determined.

Indeed, a utility of some instruments [e.g., Coach Analysis Intervention System (CAIS) Cushion et al., 2012a] is the ability to allow focus on specific aspects of coach behavior, using one or two elements of primary behavior with associated detail of secondary behavior according to need (Cushion et al., 2012a). In this way multiple “sub-instruments” within a tool can be created that focus on specific aspects of coaching practice or coach development (i.e., Mason et al., 2020). This approach also lends itself to live coding giving the coach instant feedback and allowing the live coding to be linked back and synched with recorded video footage for further future or fuller analysis (Cushion et al., 2012a). By selecting components from an existing validated instrument, research has demonstrated that systematic observation can become a central part of coaches' repertoire for reflective practice, this is both in action and on action (Partington et al., 2015), which gives coach developers' a repertoire for supporting coach learning (Cope et al., 2021). As noted in this article, an established body of evidence supporting CAR approaches is emerging whereby coaches and coach developers become researchers of their own practice. It is our contention that systematic observation is well-placed to further support and stimulate these collaborative opportunities. Coaching and its future development can be informed by research programs embedded in practice that are theoretically and empirically sophisticated where the analysis of coaching practice in real settings (in collaboration with coaches) provides the tools to better comprehend coaches' and athletes' individual and collective work (Cushion, 2007; Lyle and Cushion, 2017).

## Data availability statement

The original contributions presented in the study are included in the article/supplementary materials, further inquiries can be directed to the corresponding author/s.

## Author contributions

EC designed and conceptualized the manuscript and wrote the initial drafts. CC wrote sections of the paper and edited the full manuscript. SH and MP read, revised, and approved the submitted manuscript. All authors contributed to the article and approved the submitted version.

## Conflict of interest

The authors declare that the research was conducted in the absence of any commercial or financial relationships that could be construed as a potential conflict of interest.

## Publisher's note

All claims expressed in this article are solely those of the authors and do not necessarily represent those of their affiliated organizations, or those of the publisher, the editors and the reviewers. Any product that may be evaluated in this article, or claim that may be made by its manufacturer, is not guaranteed or endorsed by the publisher.
